# Dietary Sphingomyelin Lowers Hepatic Lipid Levels and Inhibits Intestinal Cholesterol Absorption in High-Fat-Fed Mice

**DOI:** 10.1371/journal.pone.0055949

**Published:** 2013-02-07

**Authors:** Rosanna W. S. Chung, Alvin Kamili, Sally Tandy, Jacquelyn M. Weir, Raj Gaire, Gerard Wong, Peter J. Meikle, Jeffrey S. Cohn, Kerry-Anne Rye

**Affiliations:** 1 Nutrition and Metabolism Group, Heart Research Institute, Sydney, New South Wales, Australia; 2 Lipid Research Group, Heart Research Institute, Sydney, New South Wales, Australia; 3 Metabolomics Laboratory, Baker IDI Heart and Diabetes Institute, Melbourne, Victoria, Australia; 4 Department of Medicine, University of Sydney, Sydney, New South Wales, Australia; 5 Department of Medicine, University of Melbourne, Melbourne, Victoria, Australia; University College Dublin, Ireland

## Abstract

Controlling intestinal lipid absorption is an important strategy for maintaining lipid homeostasis. Accumulation of lipids in the liver is a major risk factor for metabolic syndrome and nonalcoholic fatty liver disease. It is well-known that sphingomyelin (SM) can inhibit intestinal cholesterol absorption. It is, however, unclear if dietary SM also lowers liver lipid levels. In the present study (i) the effect of pure dietary egg SM on hepatic lipid metabolism and intestinal cholesterol absorption was measured with [^14^C]cholesterol and [^3^H]sitostanol in male C57BL/6 mice fed a high-fat (HF) diet with or without 0.6% wt/wt SM for 18 days; and (ii) hepatic lipid levels and gene expression were determined in mice given a HF diet with or without egg SM (0.3, 0.6 or 1.2% wt/wt) for 4 weeks. Mice supplemented with SM (0.6% wt/wt) had significantly increased fecal lipid and cholesterol output and reduced hepatic [^14^C]cholesterol levels after 18 days. Relative to HF-fed mice, SM-supplemented HF-fed mice had significantly lower intestinal cholesterol absorption (−30%). Liver weight was significantly lower in the 1.2% wt/wt SM-supplemented mice (−18%). Total liver lipid (mg/organ) was significantly reduced in the SM-supplemented mice (−33% and −40% in 0.6% wt/wt and 1.2% wt/wt SM, respectively), as were triglyceride and cholesterol levels. The reduction in liver triglycerides was due to inactivation of the LXR-SREBP-1c pathway. In conclusion, dietary egg SM has pronounced hepatic lipid-lowering properties in mice maintained on an obesogenic diet.

## Introduction

Controlling lipid absorption in the intestine is an important strategy for maintaining lipid homeostasis. Hepatic steatosis is a significant risk factor for many metabolic and cardiovascular diseases, such as atherosclerosis and nonalcoholic fatty liver disease (NAFLD). Reducing cholesterol absorption is considered to be atheroprotective [1], [2], and is associated with reduced risk of NAFLD in mice [3].

This study asks if dietary supplementation with sphingomyelin (SM) reduces cholesterol absorption in mice fed a high-fat (HF) western-type diet. SM is a sphingolipid found in foods such as milk and eggs. Purified SM has been shown to inhibit cholesterol absorption in a number of acute *in vitro* [4] and *in vivo* [5]–[8] studies. A recent study reported plasma and hepatic lipid lowering effects in Zucker fatty rats fed a low-fat chow diet supplemented with chicken skin-derived SM for 6 weeks [9]. However, the lack of dietary fat in the chow diet used in that study did not address the question as to whether long-term dietary SM supplementation affects cholesterol absorption inhibition. Nor was it able to correlate the inhibition of intestinal cholesterol absorption to lipid-lowering effects [9].

Mice fed an obesogenic diet containing high levels of butter fat and cholesterol develop features similar to those of humans with the metabolic syndrome (increased body weight, hepatic steatosis and insulin resistance) [10]. Previous work from our laboratory has shown that dietary supplementation with phospholipids from different sources such as milk, krill, egg and soy can reduce plasma and liver lipid levels [3], [11]–[13]. In this study, our aim was to determine whether dietary SM supplementation inhibits intestinal cholesterol absorption in mice fed an obesogenic diet, and to determine whether this effect is linked to a beneficial effect on hepatic lipid levels.

## Materials and Methods

### Animals and diets

All of the animal studies were approved by the Animal Welfare Committee of the Sydney South West Area Health Service.

Four to five-week-old male C57BL/6 mice were obtained from Monash University, Melbourne, VIC. They were housed in standard cages (5 mice per cage) at a constant temperature of 21°C with a 12 h light/dark cycle and *ad libitum* access to a normal chow diet and water. After 1 week of acclimatization, the mice were randomly assigned to four groups (n = 10/group). The animals were given a high-fat semi-purified diet with (HFSM) or without (SM) egg SM (99% pure, Lipoid, Ludwigshafen, Germany) for 18 days (Study 1) or for 4 weeks (Study 2).

The high-fat diet contained 21% (wt/wt) butterfat and 0.15% (wt/wt) cholesterol. The composition was as follows: casein, 195 g/kg; DL-methionine, 3 g/kg; sucrose 341 g/kg; wheat starch, 154 g/kg; cellulose, 50 g/kg; clarified butter, 210 g/kg; calcium carbonate, 17.1 g/kg; sodium chloride, 2.6 g/kg; potassium citrate, 2.6 g/kg; potassium dihydrogen phosphate, 6.9 g/kg; potassium sulphate, 1.6 g/kg; AIN93G trace minerals, 1.4 g/kg; choline chloride (65%), 2.5 g/kg; vitamins, 10 g/kg; cholesterol, 1.5 g/kg. The SM supplement contained at least 99% (wt/wt) sphingomyelin and the following minor components: phosphatidylcholine (∼0.5%), lysophosphatidylcholine (∼0.2%), triglycerides (∼1.0%) and free fatty acids (∼0.2%). The main phospholipid species in the SM supplement were palmitic acid (16∶0) and stearic acid (18∶0), which contributed a maximum of 89% and 6%, respectively.

In Study 1, the high-fat diet was supplemented with SM 0.6% wt/wt. In Study 2, the high-fat diet was supplemented with 0.3%, 0.6% or 1.2% SM (wt/wt). Food consumption was recorded three times per week and body weight was monitored weekly.

### Intestinal cholesterol absorption

In order to investigate the ability of SM to inhibit intestinal cholesterol absorption, two groups of mice (n = 10/group) were fed a high-fat diet with or without SM (0.6% wt/wt) for 18 days (Study 1). At 14 days after commencing the diet, olive oil (200 µl) containing 1 µCi [^14^C]cholesterol and 1 µCi [^3^H]sitostanol, a sterol that is minimally absorbed by the intestine [14], was administered by intragastric gavage under light methoxyflurane anaesthesia. One animal from the SM-supplemented group did not survive the gavage and was excluded from the analysis. The mice were then transferred into individual cages with perforated-metal floors. Feces was collected daily for 4 days and stored at −80°C until analysis. Mice were given *ad libitum* access to their respective diets during this collection period so that the total length of the dietary intervention was 18 days. At the end of the fecal collection period, the mice were anaesthetized with methoxyflurane and exsanguinated by heart puncture.

At sacrifice, livers were immediately excised, weighed and divided into 100–150 mg sections for storage at −80°C until analysis for lipid and radiolabel content. Fecal samples were combined, dried at 60°C for 24 hours then ground to a fine powder. Total lipid content in feces and the pre-gavage radioactive cholesterol-sitostanol-olive oil mixture was analyzed by extracting lipids from 100 mg of the fecal powder or 10 µl aliquots of the olive oil mixture with 1.2 ml of chloroform∶methanol (2∶1, v/v) [15]. The lipid extracts were dried under nitrogen, saponified in 1 ml of 2 M sodium hydroxide∶methanol (1∶1, v/v) and incubated at 60°C for 1 hour. The labelled sterols were then extracted into diethyl ether (2 ml). Duplicate aliquots (500 µl) of the organic phase were dried by evaporation, scintillation fluid (5 ml) was added and the samples were counted. The percentage of cholesterol absorption was calculated using the ratio of ^14^C and ^3^H counts in the olive oil mixture and feces according to the fecal dual-isotope method [16].
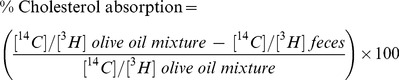
(1)The total cholesterol and triglyceride levels in the liver and feces were measured biochemically as described below.

### Hepatic lipid analysis

To investigate the effect of SM supplementation on hepatic lipid profile, four groups of mice (n = 10/group) were given high-fat diet without or with 0.3%, 0.6% and 1.2% (wt/wt) SM for 4 weeks (Study 2). The animals were fasted for 12 hours before sacrifice. Two high-fat fed animals were subsequently excluded from the study because they failed to thrive. Livers were excised immediately after sacrifice, weighed and sectioned (100–150 mg/section) for storage at −80°C for subsequent lipid analysis, or added to RNA*later*® solution (Albion, Austin, TX) for gene expression analysis.

Total liver and fecal lipids were determined gravimetrically after extraction with cholororm∶methanol (2∶1, v/v) [15]. Liver and fecal total cholesterol and triglyceride levels were quantified enzymatically after solubilization in isopropanol using CHOD-PAP and GPO-PAP kits (Roche Diagnostics), respectively. Isopropanol-solubilized total liver phospholipids were measured using a Wako Phospholipids C kit (Wako Pure Chemicals, Osaka, Japan).

### Lipids and ceramide analysis by ESI-MS/MS

Lipids were extracted from liver homogenate (25 µg of protein) with a 20 volume excess of chloroform∶methanol (2∶1, v/v), following the addition of an internal standard mixture containing 100 pmol each of ceramide (Cer) 17∶0, deuterated glucosylceramide (a monohexosylceramide) 16∶0 (*d3*), deuterated lactosylceramide (a dihexosylceramide) 16∶0 (*d3*) (Matreya Inc., Pleasant Gap, USA), phosphatidylcholine (PC) 13∶0/13∶0, phosphatidylethanolamine (PE) 17∶0/17∶0, phosphatidylserine (PS) 17∶0/17∶0 and sphingomyelin 12∶0 (Avanti Polar Lipids, Alabaster, USA), together with 1000 pmol each of deuterated unesterified cholesterol *(d7)* (Avanti Polar Lipids, Alabaster, USA) and deuterated cholesteryl ester (CE) 18∶0 *(d6)* (CDN Isotopes, Pointe-Claire, Quebec, Canada). The samples were vortexed, rotated for 10 minutes, sonicated for 30 minutes and left to stand at room temperature for a further 20 minutes. The extracts were then centrifuged at 13,000×g for 10 minutes, and supernatants were transferred to a 96-well plate and dried under nitrogen at 40°C. Lipids were redissolved in water-saturated butanol (50 µl) with sonication (10 min) followed by the addition of methanol (50 µl) containing 10 mM NH_4_COOH. Samples were analysed by electrospray ionisation-tandem mass spectrometry using a PE Sciex API 4000 Q/TRAP mass spectrometer with a turbo-ionspray source and Analyst 1.5 data system. Prior liquid chromatographic separation was performed on a Zorbax C18, 1.8 um, 50×2.1 mm column at 300 µl/min using the following gradient conditions; 100% solvent A to 0% solvent A over 8.0 min followed by 2.5 min at 0% solvent A, a return to 100% solvent A over 0.5 min then 3.0 min at 100% solvent A prior to the next injection. Diglycerides and triglycerides were separated on an isocratic (85% B) system over 6 minutes. Solvents A and B consisted of tetrahydrofuran∶methanol∶water in the ratios (30∶20∶50, v/v/v) and (75∶20∶5, v/v/v), respectively, plus 10 mM NH_4_COOH.

Individual lipid species were quantified using scheduled multiple-reaction monitoring (MRM) in positive ion mode. Individual lipid species monitored were the major species (greater than 1% of total) identified in human plasma. MRM experiments were based on product ion of *m/z* 264 [sphingosine–H_2_O]^+^ for Cer, GC and DHC, *m/z* 184 [phosphocholine]^+^ for SM, *m/z* 369 [cholesterol-H_2_O]^+^ for cholesterol and CE. Each ion pair was monitored for 10–50 ms with a resolution of 0.7 amu at half-peak height and averaged from continuous scans over the elution period. Lipid concentrations were calculated by relating the peak area of each species to the peak area of the corresponding internal standard. Total lipids of each class were calculated by summing the individual lipid species.

### Gene expression analysis

Total RNA was isolated from liver samples using an RNeasy kit (Qiagen, Melbourne, Australia). RNA (100 ng) was reverse transcribed into cDNA using random primers provided with the iScript cDNA Synthesis Kit (Bio-Rad, Sydney, Australia). mRNA levels were measured by real time PCR. Selected genes were amplified using iQ SYBR Green Supermix (Bio-Rad) in an iCycler system (Bio-Rad) with 20 pmol of both forward and reverse primers (Table S1). PCR conditions were as follows: 1 cycle of 95°C for 3 min, 40 cycles of 95°C for 30 sec, 55–60°C for 3 sec and 72°C for 30 sec, followed by 1 cycle of 95°C for 1 min. Purity of the PCR products was assessed by melt curve analysis. Relative gene expression was calculated by normalizing cycle threshold (Ct) values for genes of interest with Ct values for cyclophilin using the ΔΔCt method.

### Statistical analysis

The results are reported as means±SEM. Graphpad Prism (version 4.0c, GraphPad Software, Inc.) was used for statistical analyses. Significant differences between the HF and HFSM groups were assessed by either one-way ANOVA or Student's *t*-test (homoscedastic, two-tailed). Pearson correlation coefficients (r) were determined to assess the relationship between parameters, with *P*<0.05 considered to be statistically significant. The p-values were corrected for multiple comparisons (*P_corrected_*) using the Benjamini-Hochberg [17] approach for genes, lipids and lipid classes separately, *P*_corrected_<0.05 was considered to be statistically significant.

Hierarchical clustering based on correlation coefficients derived from all significant genes and lipids between the HF and HFSM groups was performed using the MATLAB 2012b Bioinformatics Toolbox.

A relevance network was created using Cytoscape [18] (version 2.2.2) where the nodes are genes and lipids and an edge is defined between genes (green) or genes and lipids (blue) for |r|>0.7 and p-value<0.05.

## Results

### Study 1: Inhibition of intestinal cholesterol absorption and reduction in hepatic lipids by dietary SM

Dietary supplementation with SM for 18 days did not affect total body weight (data not shown). Mice supplemented with SM had slightly smaller livers than the control animals (1.13±0.05 v 1.21±0.05 g) and their liver/body weight ratio was also less (4.49±0.23 v 4.95±0.18). These differences did not reach statistical significance. The livers of the high fat-fed mice supplemented with SM contained significantly less total lipid (−37%, *P*<0.05), cholesterol (−55% *P*<0.001) and triglyceride (−49%, *P*<0.05) than the livers of the high fat-fed control mice (Table 1). They also contained significantly lower levels of intestinally-derived [^14^C]cholesterol. There was no difference in hepatic [^3^H]sitostanol levels between the two groups.

**Table 1:** Effect of dietary SM on liver and fecal lipids, and on intestinal cholesterol absorption in high-fat fed mice.

	HF	HFSM 0.6%	*Difference*
	(n = 10)	(n = 9)	
Liver			
Total lipid (mg/organ)	105.3±12.8	66.8±8.9*	*−37%*
Cholesterol (µmol/organ)	31.4±2.9	14.1±1.7***	*−55%*
Triglyceride (µmol/organ)	99.3±15.9	50.7±9.4*	*−49%*
[^14^C]cholesterol (dpm/organ)	7531±1314	1725±255**	*−77%*
[^3^H] sitostanol (dpm/organ)	341±35	285±30	*−17%*
Feces			
Total lipid (mg/mouse/day)	10.4±1.3	15.4±2.3	*+48%*
Cholesterol (µmol/mouse/day)	9.2±0.7	12.7±0.7**	*+37%*
Triglyceride (µmol/mouse/day)	2.6±1.0	3.3±1.0	*+27%*
[^14^C]cholesterol (dpm/mouse/day)	9659±601	12604±1004*	*+30%*
[^3^H] sitostanol (dpm/mouse/day)	19593±1571	20018±2069	*+2%*
Cholesterol absorption (%)	52.4±2.6	36.8±3.9**	*−30%*

Male C57BL/6 mice were fed a high-fat diet without (HF) or with 0.6% wt/wt SM (HFSM 0.6%) for 18 days. The mice were gavaged with olive oil containing radioactively labelled cholesterol and sitostanol on day 14 and sacrificed on day 18. Feces were collected on days 14–18. The livers were excised at sacrifice. Fecal and hepatic lipids were quantified as described in “Methods”. Results represent means±SEM. Significant differences between HF and HFSM groups were determined by Student's t-test:

* *P*<0.05,

** *P*<0.01,

*** *P*<0.001.

**Figure 1:**
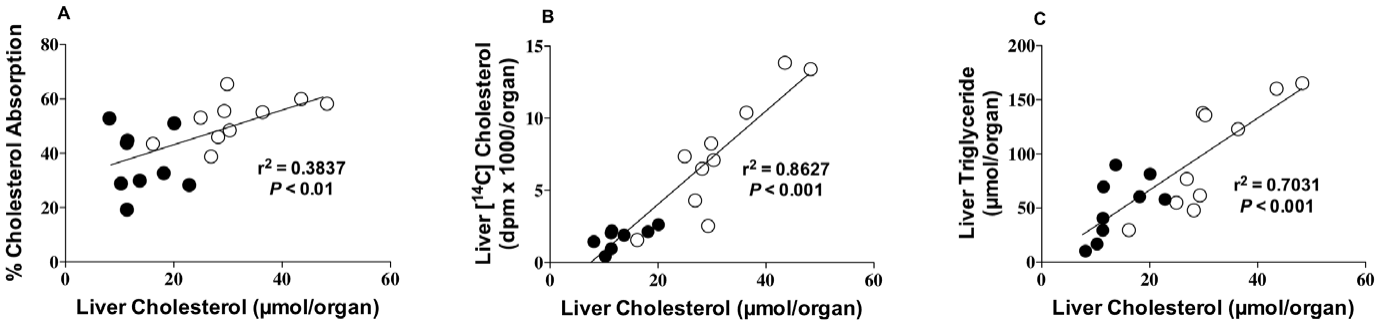
Relationship between liver cholesterol and A) percentage of intestinal cholesterol absorbed; B) liver [^14^C]cholesterol and C) liver triglyceride levels in mice fed a high-fat diet or a high-fat diet supplemented with 0.6% (wt/wt) sphingomyelin. Each point represents data for an individual animal. Open circles represent high-fat-fed mice; filled circles represent high-fat fed animals supplemented with SM.

SM supplementation was associated with a 30% increase in fecal [^14^C]cholesterol content (*P*<0.05) (Table 1). Similar levels of fecal [^3^H]sitostanol in both groups of animals indicated that comparable amounts of radioactive sterol were administered on day 14. Using these data, cholesterol absorption was found to be significantly less in mice supplemented with SM than in the mice fed a HF diet without SM supplementation (−30%, *P*<0.01). This was also associated with higher amounts of cholesterol and total lipid in the feces of the SM-fed animals. Linear regression analysis showed that liver cholesterol in the HF- and HFSM-fed animals was significantly correlated with percentage of intestinal cholesterol absorption (r^2^ = 0.38, *P*<0.01) (Fig. 1A), and with levels of intestinally-derived cholesterol in the liver (r^2^ = 0.86, *P*<0.001) (Fig. 1B). Liver cholesterol levels were also correlated with liver triglyceride levels (r^2^ = 0.70, *P*<0.001) (Fig. 1C). Separate correlation analyses for the control and SM-treated groups were also performed. Liver cholesterol levels correlated significantly with % cholesterol absorption, liver [^14^C]cholesterol content and liver triglyceride levels in the control group. The same correlations for the SM-treated group were all poor because cholesterol absorption was very low regardless of liver cholesterol levels. This is consistent with dietary SM being highly efficacious in terms of lowering hepatic lipid levels and inhibiting intestinal cholesterol absorption, and probably reflects maximal inhibition of cholesterol absorption by the SM dose used in the study. In order to display the relationships of interest over a wider range, the combined data sets are shown in Figure 1.

### Study 2: Effect of dietary SM on hepatic lipid metabolism

In order to further investigate the effect of dietary SM on liver lipid levels, a second study was carried out in which high-fat fed mice received dietary supplementation with 0.3%, 0.6% and 1.2% (wt/wt) SM for 4 weeks (Study 2). The high fat-fed control mice, and the mice fed a high fat diet supplemented with SM, all had similar body weight gain and food intake throughout the study (Table 2). The liver weights of the animals supplemented with SM decreased in a dose-dependent manner. Mice fed 0.6% and 1.2% (wt/wt) SM had liver weights that were 12% (*P*<0.05) and 18% (*P*<0.001) lower, respectively, than what was observed for the HF animals (Table 2). Their liver/body weight ratios were also lower, but this reached statistical significance only in the animals that received supplementation with 1.2% (wt/wt) SM (*P*<0.05).

**Table 2:** Body weight, liver weight and food intake of mice fed a high-fat diet with or without SM.

	HF	HFSM 0.3%	HFSM 0.6%	HFSM 1.2%
	(n = 8)	(n = 10)	(n = 10)	(n = 10)
Initial body wt (g)	17.25±0.63	17.26±0.43	16.78±0.78	16.71±0.55
Final body wt (g)	25.95±0.94	25.46±0.51	24.00±0.54	25.07±0.59
Wt gain (g)	8.71±1.07	8.19±0.80	7.22±0.78	8.36±0.68
Liver wt (g)	1.05±0.04	0.99±0.02	0.92±0.02*	0.86±0.03***
Liver wt/body wt (g/100 g)	4.08±0.21	3.92±0.12	3.85±0.09	3.44±0.13*
Food intake (g/mouse/day)	3.44±0.14	3.30±0.14	3.29±0.16	3.38±0.12

Male C57BL/6 mice were fed a high-fat (HF) diet without or with SM (HFSM) (0.3%. 0.6% or 1.2% wt/wt) and sacrificed after 4 weeks. Body and liver weights were recorded. Liver lipids were quantified as described in “Methods”. Values represent means±SEM. Significant differences between HF and HFSM groups were determined by one-way ANOVA:

* *P*<0.05;

*** *P*<0.001.

**Figure 2:**
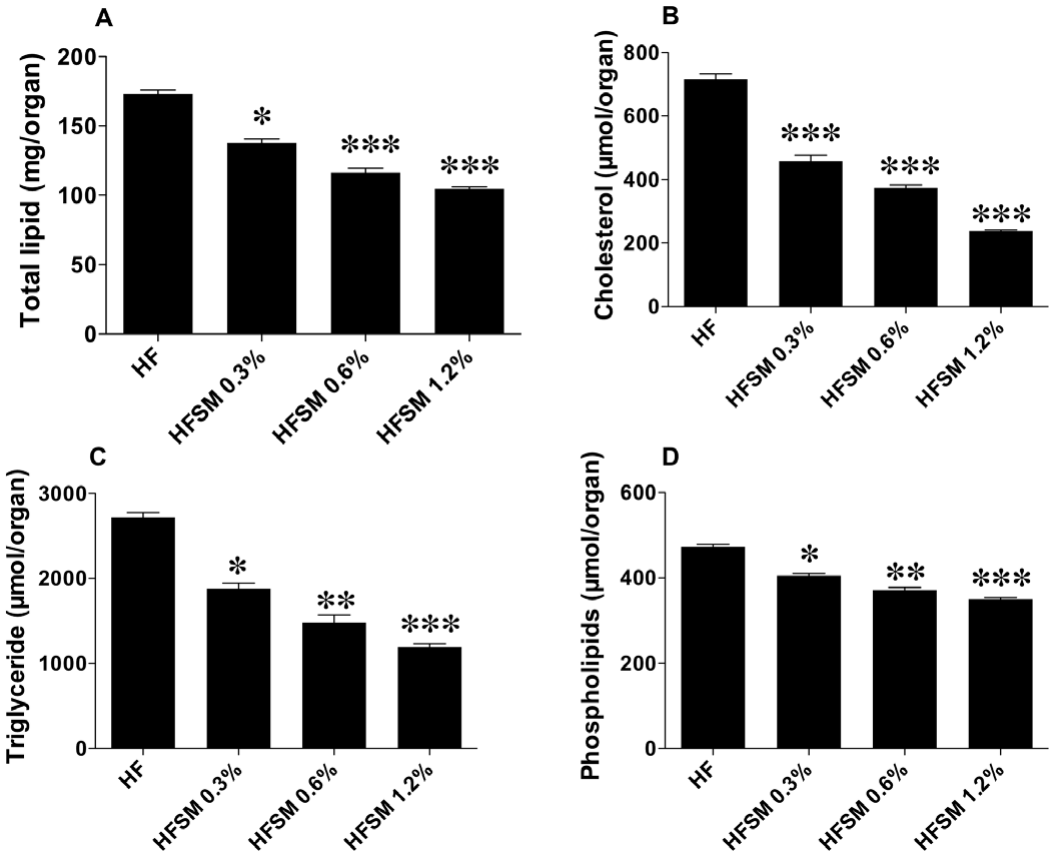
Total liver lipid (A), liver cholesterol (B), liver triglyceride (C) and liver phospholipid levels (D) in mice fed a high-fat diet or a high-fat diet supplemented with varying doses of egg sphingomyelin. Bars represent means±SEM (*n* = 8–10 mice per group). Differences between groups were determined by one-way ANOVA: *P<0.05, **P<0.01, ***P<0.001.

Total hepatic lipid, cholesterol, triglyceride and phospholipid levels were also reduced in a concentration-dependent manner in the SM-supplemented animals (Fig. 2). Total hepatic lipid levels (mg/organ) were decreased by 20%, 33% and 40% in the 0.3%, 0.6% and 1.2% (wt/wt) SM-supplemented groups, respectively (Fig. 2A). Hepatic cholesterol levels were decreased by 36%, 48% and 67% (Fig. 2B); while hepatic triglyceride levels were decreased by 31%, 46% and 56%, respectively (Fig. 2C). Hepatic phospholipid levels were also lower in the SM-supplemented animals, but not to the same extent (14%, 21% and 24%, respectively, Fig. 2D).

Individual and total lipid concentrations in the livers of the high fat-fed control mice, and the mice fed a high fat diet supplemented with SM were also determined using a p-value corrected for multiple comparisons as described in the Methods. These results are shown in Tables 3 and 4 (Table S2 for complete results).

**Table 3:** Cholesterol, triglyceride, sphingomyelin and ceramide levels in livers of mice fed a high-fat diet supplemented with SM.

	HF	HFSM 0.6%	Difference
	(n = 8)	(n = 10)	
	*nmol/mg protein*	*nmol/mg protein*	
Total cholesterol	269.9±36.8	122.1±16.2†	*−55%*
Cholesteryl ester	239.0±36.0	97.0±13.7†	*−59%*
Unesterfied cholesterol	31.0±3.7	25.1±3.3	*−19%*
Triglyceride	554.8±58.0	305.9±41.1†	*−45%*
Diglyceride	103.7±10.4	75.6±10.6	*−27%*
Sphingomyelin	4.3±0.2	4.0±0.3	*−6%*
Total ceramide	1.2±0.1	1.0±0.1	*−15%*
Total monohexosylceramide	0.67±0.09	0.57±0.05	*−14%*
Total dihexosylceramide	0.04±0.006	0.03±0.004	*−28%*

Male mice were fed a high-fat diet (HF) without or with 0.6% wt/wt SM (HFSM) for 4 weeks. Liver lipids were quantified as described in “Methods”. Values represent means±SEM. Percentage difference between groups is given in italics. Significant differences between the HF and HFSM groups were determined by Student's t-test:

† *P_corrected_*<0.05.

**Table 4:** Levels of significantly different lipids in livers of mice fed a high-fat diet supplemented with SM.

	HF	HFSM 0.6%	Difference
	(n = 8)	(n = 10)	
Lipid	*nmol/mg protein*	*nmol/mg protein*	
Cer 24∶1	460±39	323±24†	*−30%*
SM 41∶2	484±31	339±30†	*−30%*
PC 38∶3	3543±278	2421±205†	*−32%*
PC(O-36∶3)	329±37	196±13†	*−40%*
LPC 20∶1	10±1	5±1†	*−51%*
PE(O-40∶6)	328±16	252±14†	*−23%*
PE(P-38 ∶5)	41±5	26±2†	*−38%*
PE(P-40∶6)	8±1	5±0†	*−36%*
BMP 18∶1/18∶1	715±93	389±39†	*−46%*
CE 14∶0	4610±553	1602±230†	*−65%*
CE 15∶0	2619±472	812±191†	*−69%*
CE 16∶0	33579±4726	15554±2117†	*−54%*
CE 16∶1	138641±22823	51330±7417†	*−63%*
CE 16∶2	742±127	256±55†	*−65%*
CE 17∶0	4471±653	1899±309†	*−58%*
CE 17∶1	3985±676	1628±266†	*−59%*
CE 18∶1	39083±5215	17560±2439†	*−55%*
CE 18∶3	930±123	454±84†	*−51%*
CE 22∶1	180±20	53±16†	*−71%*
TG 14∶0/18∶0/18∶1	258±30	142±19†	*−45%*
TG 14∶1/18∶0/18∶2	1767±242	819±170†	*−54%*
TG 14∶1/18∶1/18∶1	12806±1778	5940±923†	*−54%*
TG 15∶0/18∶1/18∶1	2401±235	1278±178†	*−47%*
TG 16∶0/16∶1/18∶1	97150±10256	55317±7414†	*−43%*
TG 16∶0/17∶0/18∶2	6461±617	3685±487†	*−43%*
TG 16∶0/18∶0/18∶1	2169±198	1425±157†	*−34%*
TG 16∶0/18∶1/18∶1	130524±10856	73831±9649†	*−43%*
TG 16∶0/18∶1/18∶2	18175±1085	11639±1557†	*−36%*
TG 16∶1/16∶1/18∶0	644±92	337±54†	*−48%*
TG 16∶1/16∶1/18∶1	23860±3271	11337±1678†	*−52%*
TG 16∶1/17∶0/18∶1	11325±1088	6076±814†	*−46%*
TG 16∶1/18∶1/18∶1	30022±3220	13082±1765†	*−56%*
TG 16∶1/18∶1/18∶2	8095±741	4292±595†	*−47%*
TG 17∶0/18∶1/18∶1	2998±280	1462±192†	*−51%*
TG 18∶0/18∶1/18∶1	2142±269	876±101†	*−59%*
TG 18∶0/18∶2/18∶2	1026±76	581±84†	*−43%*
TG 18∶1/18∶1/18∶1	31499±3672	11295±1408†	*−64%*
TG 18∶1/18∶1/18∶2	1261±122	525±74†	*−58%*
TG 18∶1/18∶1/20∶4	496±27	303±37†	*−39%*
TG 18∶1/18∶1/22∶6	828±56	514±67†	*−38%*
TG 18∶1/18∶2/18∶2	1786±119	1013±138†	*−43%*

Male mice were fed a high-fat (HF) diet without or with 0.6% wt/wt SM (HFSM) for 4 weeks. Liver lipids were quantified as described in “Methods”. Values represent means±SEM. Percentage difference between groups is given in italics. Significant differences between the HF and HFSM groups were determined by Student's t-test:

† *P_corrected_*<0.05. Cer: Ceramide; SM Sphingomyelin; PC: Phosphatidylcholine; LPC: Lysophosphatiylcholine; PE: Phosphatidylethanolamine; BMP: Bis(monoacylglycero)phosphate; CE: Cholesteryl ester; TG: Triglyceride.

Total liver cholesterol levels were significantly lower (−55%) in the SM-supplemented mice (Table 3). Fifteen lipids of the CE class were detected by ESI-MS/MS (Table S2), ten of which were significantly lower in the livers of the animals that received SM (Table 4). Total hepatic CE was reduced by 59% in the SM-supplemented mice (Table 3). Unesterified cholesterol, levels were also lower in SM-supplemented animals (−19%), but this did not reach statistical significance.

Total triglyceride (TG) and, diglyceride (DG) levels were also decreased by 45% (*P*<0.05) and 27% (n.s.) respectively. Thirty-nine lipids of the TG class were detected (Table S2) and 22 of them were significantly lower in the SM-supplemented mice (Table 4). These included TG 16∶1/18∶1/18∶1 (−43%), TG 18∶1/18∶1/18∶1 (−64%), and TG 18∶1/18∶1/18∶2 (−58%). Although dietary supplementation with SM also lowered DG levels, these differences were not significant after correction for multiple comparisons (Table S2).

Dietary supplementation with SM did not significantly affect hepatic SM levels (Table 3). Total ceramide (Cer) levels were also unchanged. However, Cer 24∶1 was significantly lower in the SM-supplemented (Table 4) group and Cer 16∶0 also showed a non-significant negative trend after correction for multiple comparisons (Table S2). Similar trends were observed in both monohexosylceramides and dihexosylceramides, with the 24∶1 and 16∶0 species showing negative trends in the SM-supplemented group (Table 3, Table S2).

**Figure 3:**
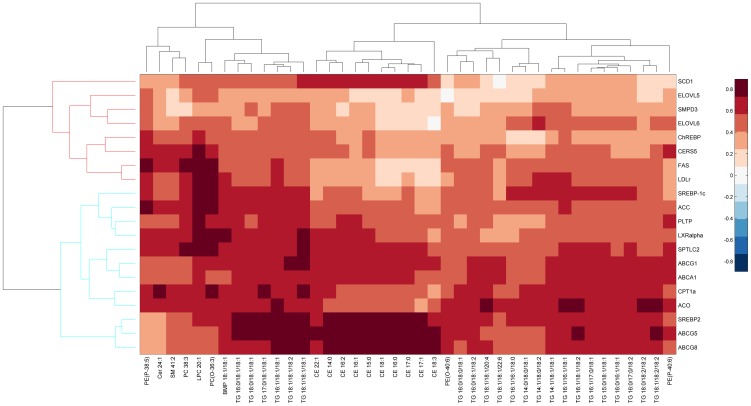
Hierarchical clustering based on correlation coefficients derived from all significant changes in gene expression and lipid levels between high fat-fed mice and high fat-fed, SM-supplemented mice.

**Figure 4:**
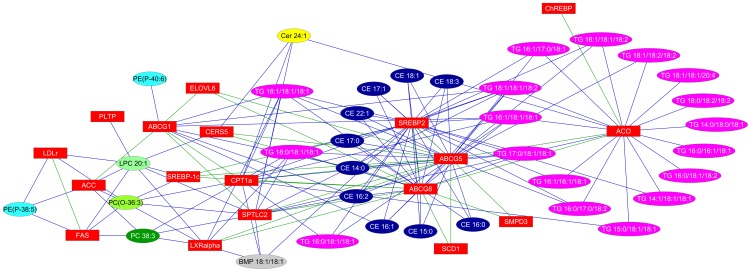
A relevance network of significantly different genes and lipids between high fat-fed mice and high fat-fed, SM-supplemented mice.

Messenger RNA levels of genes involved in liver fatty acid and cholesterol metabolism are shown in Table 5. The hierarchical clustering of correlations of genes and lipids which were significantly different in the two groups is shown in Figure 3. The relevance network of these genes and lipids is shown in Figure 4. Strikingly, mRNA levels for all four ATP-binding cassette transporter genes, ABCA1, ABCG1, ABCG5 and ABCG8, that were measured were significantly lower in the SM-supplemented mice (−28%, −57%, −44% and −32% respectively, *P*<0.001 for all). These genes were also positively correlated with several CE and TG lipids (Figure 3). The ABCG5/8 and SREBP-2 genes were also positively correlated, with the significantly altered CEs (Figure 3).

**Table 5:** Hepatic mRNA levels in mice fed a high-fat diet with and without SM supplementation.

	HF	HFSM 0.6%	Difference
	(n = 8)	(n = 10)	
***Cholesterol efflux***			
ABCA1	1.00±0.03	0.72±0.03†	−28%
ABCG1	1.03±0.09	0.44±0.02†	−58%
ABCG5	1.04±0.11	0.58±0.03†	−44%
ABCG8	1.01±0.56	0.69±0.02†	−31%
***Cholesterol uptake***			
LDLr	1.02±0.09	0.74±0.04†	−28%
SR-B1	1.01±0.05	0.90±0.05	−11%
***Cholesterol synthesis***			
HMGCoAR	1.02±0.07	1.02±0.06	0%
HMGCoASyn	1.05±0.13	0.82±0.08	−22%
***Other LXR-regulated gene***			
PLTP	1.07±0.13	0.56±0.03†	−47%
***Fatty Acid Synthesis***			
FAS	1.04±0.11	0.65±0.06†	−38%
ACC	1.04±0.11	0.54±0.04†	−48%
ELOVL5	1.06±0.14	0.62±0.06†	−42%
ELOVL6	1.03±0.09	0.71±0.06†	−31%
SCD1	1.04±0.11	0.70±0.08†	−33%
***Triglyceride Synthesis***			
DGAT2	1.04±0.11	0.99±0.04	−4%
***Fatty Acid Oxidation***			
ACO	1.03±0.09	0.47±0.06†	−54%
CYP4A10	1.02±0.08	0.83±0.07	−19%
CPT1a	1.01±0.06	0.78±0.04†	−23%
MCAD	1.06±0.14	0.89±0.07	−16%
VLCAD	1.05±0.12	0.80±0.04	−23%
***De novo ceramide synthesis***			
SPTLC2	1.02±0.07	0.72±0.02†	−30%
CERS2	1.04±0.11	0.82±0.02	−21%
CERS5	1.06±0.13	0.64±0.02†	−39%
***Ceramide generation from SM***			
SMPD1	1.01±0.05	0.94±0.04	−7%
SMPD3	0.92±0.04	0.61±0.07†	−34%
***Transcription Factor***			
LXRα	1.02±0.07	0.80±0.01†	−21%
SREBP2	1.01±0.05	0.65±0.02†	−36%
SREBP-1C	1.05±0.12	0.58±0.04†	−44%
PPARα	1.03±0.10	0.86±0.05	−17%
ChREBP	1.04±0.11	0.63±0.06†	−35%

Total RNA was extracted from livers as described in “Methods” and mRNA levels were quantified by real time PCR. Results are expressed relative to cyclophilin. Differences between groups were determined by Student's t-test:

† *P_corrected_*<0.05.

SM-supplementation decreased mRNA levels of liver X receptor α (LXRα), which transcriptionally regulates ATP-binding cassette transporter genes, by 22% (*P*<0.01). Phospholipid transfer protein (PLTP), another LXRα-regulated gene, was also significantly lower in the livers of the SM-supplemented mice. Other transcription factor mRNAs significantly reduced by SM supplementation included sterol regulatory element-binding protein-2 (SREBP-2), sterol regulatory element-binding protein-1c (SREBP-1c), and the carbohydrate response element binding protein (ChREBP). SREBP-2 was highly correlated with CE lipids while SREBP-1c was highly correlated with a number of phospholipid species (PC 38∶3, LPC 20∶1 and PC(O-36∶3)), and moderately correlated with TG lipids (Figure 3). ChREBP, though correlated with ACO which appeared to be highly co-regulated with TGs, was not strongly correlated with any lipid. The mRNA level of the low-density lipoprotein receptor (LDLr), which is responsible for cholesterol uptake by the liver, was also reduced significantly in the SM-supplemented mice (−28%) and was strongly correlated with PC(O-36∶3), PC 38∶5 and LPC 20∶1. However, other SREBP2-regulated genes, such as scavenger receptor-B1 (SR-B1), and enzymes that control cholesterol synthesis, such as 3-hydroxy-3-methylglutaryl coenzyme reductase (HMGCoA reductase) and 3-hydroxy-3-methylglutaryl coenzyme synthase (HMGCoA synthase), were not affected (Table 5).

Since LXRα regulates sterol regulatory element-binding protein-1c (SREBP-1c), which in turn controls the expression of genes responsible for fatty acid synthesis, expression of these genes was also investigated. mRNA levels for fatty acid synthase (FAS) (−38%, *P*<0.01), acetyl-CoA carboxylase (ACC) (−48%, *P*<0.001), elongation of very long chain fatty acids protein-5 (ELOVL5) (−42%, *P*<0.01), ELOVL6 (−31%, *P*<0.01) and steroyl-CoA desaturase 1 (SCD1) (−31%, *P*<0.05), were significantly reduced (Table 5). LXRα, FAS, LDLr and ACC, all were also highly correlated with LPC 20∶1, PC 38∶3 and PC(O-36∶3) lipids (Figure 3). Furthermore, SCD1 was also highly correlated with CEs. In contrast, ELOVL5/6 were only weakly correlated with TGs. No difference was observed in diglyceride acyltransferase (DGAT2) mRNA levels, a key enzyme involved in triglyceride synthesis.

We also hypothesized that genes controlling fatty acid oxidation may have been increased by SM supplementation, but no evidence to this effect was obtained. In fact mRNA levels of the fatty acid oxidation genes (acetyl-CoA oxidase (ACO), cytochrome P450 4A10 (CYP4A10), carnitine palmitoyltransferase 1a (CPT1a), medium-chain acyl-coenzyme A dehydrogenase (MCAD) and very long-chain acyl-coenzyme A dehydrogenase (VLCAD) tended to be lower in the mRNA levels mice than in the control, high fat-fed mice, as was the mRNA level of their transcription factor, peroxisome proliferator-activated receptor α (PPARα).

Hepatic mRNA levels of enzymes involved in *de novo* ceramide synthesis, as well as SM catabolism, were also quantified. Serine palmitoyltransferase, long chain base subunit 2 (SPTLC2) and ceramide synthase 5 (CERS5) were decreased by 29% (*P*<0.001) and 49% (*P*<0.01) respectively (Table 5), and were both strongly correlated to Cer 24∶1 (Figure 3). Dietary SM supplementation also down-regulated expression of sphingomyelin phosphodiesterase 3 (SMPD3), which hydrolyses SM to phosphocholine and ceramide, by 34% (*P*<0.01). However, while this was strongly correlated to both ABCG5 and ABCG8, it did not show the same strength of correlation to any ceramide, sphingomyelin or other lipid species (Figures 3 and 4).

## Discussion

The results of the present study demonstrate that dietary SM potently decreases total hepatic cholesterol and triglyceride levels and reduces intestinal cholesterol absorption. This suggests that dietary SM may lower hepatic lipid levels by inhibiting intestinal cholesterol absorption, and is consistent with our previously published studies demonstrating the ability of dietary phospholipid extracts (containing significant amounts of SM) to reduce hepatic lipid levels and intestinal cholesterol absorption in high-fat fed mice [3], [13].

At present, the mechanism for the inhibitory effect of dietary SM on cholesterol absorption is not well understood. The ability of dietary SM to inhibit cholesterol absorption has been shown previously [6]–[8], and it has been proposed that the strong interaction between cholesterol and SM [26], [27] reduces the micellar solubility of intestinal cholesterol, resulting in reduced intestinal cholesterol uptake [14], [16].

It has also been suggested that ceramide, the hydrolytic product of SM, inhibits intestinal cholesterol uptake [4]. Since the degradation of ceramide along the gastrointestinal tract is relatively slow and inefficient, it is possible that the inhibitory effect of ceramide on cholesterol absorption could be quite significant [19]. In addition, SM has been shown to inhibit secretory (type II) phospho]lipase A2 (sPLA2), which is structurally similar to pancreatic (type I) PLA2 (PPLA2) [20], [21]. PPLA2 activity is important in the hydrolysis of TG in the intestinal tract. It has been shown that the hydrolysis of PC to lysoPC facilitates the binding of lipase-colipase to TG (33–36) and that minimal TG hydrolysis is essential to stimulate intestinal cholesterol uptake [22]. It has thus been proposed that SM-mediated inhibition of pancreatic lipase may limit PC bioavailability, which reduces mixed micelle formation, and inhibits SM-mediated intestinal cholesterol absorption [7], [23]. It is interesting to note in the present study that total lipids, and not just cholesterol, were increased in the feces of the SM-supplemented mice (Table 1). Triglyceride itself was not significantly increased, however, and there was no evidence of severe fat malabsorption (diarrhoea or a marked reduction in body weight). The effects of dietary SM on the intestinal absorption of other lipids thus deserves further investigation.

The present study shows that lower hepatic cholesterol levels were strongly associated with reduced intestinal cholesterol absorption. Decreased flux of intestinal cholesterol to the liver is supported by the analysis of hepatic gene expression, whereby LXRα-regulated genes involved in hepatic cholesterol export, such as ABCA1, ABCG1, ABCG5 and ABCG8 [24], were down-regulated in the SM-supplemented mice. Genes responsible for hepatic cholesterol uptake, such as LDLr and SR-B1, and genes involved in cholesterol synthesis, such as HMGCoA reductase and HMGCoA synthase, by contrast, tended to be lower or were unchanged.

The observed reduction in hepatic cholesterol levels was strongly associated with lower levels of hepatic triglyceride in the present study. We hypothesize that reduced hepatic triglyceride levels in SM-supplemented mice was a consequence of reduced LXRα activity through ATP-binding cassette transporter genes, ABCA1, ABCG1, ABCG5 and ABCG8. We postulate that this may have been due to reduced availability of oxysterols, which are known to activate LXRα expression. Reduced LXRα activity has been reported to significantly decrease the expression of SREBP-1c, FAS, ACC and SCD-1 in mice [25], [26] and in hepatoma cells [27]. This is in line with our gene expression analysis showing down-regulation of SREBP-1c and the fatty acid synthesis genes FAS, ACC, ELOVL5, ELOVL6 and SCD-1 in SM-supplemented animals. Somewhat surprisingly, PPARα expression was not affected by SM-supplementation, and the expression of various PPARα-regulated genes involved in fatty acid oxidation such as ACO, CPT1a and VLCAD, were reduced, rather than increased. The current results are therefore consistent with a dietary SM-induced reduction in hepatic TG levels being associated with suppression of the LXR-SREBP-1c regulated fatty acid synthesis pathway, but not increased fatty acid oxidation. This is also supported by significant correlations between hepatic TG levels and the LXR-SREBP-1c regulated genes.

A recent study published by Yunoki *et al.* has demonstrated that feeding leptin-resistent Zucker rats a normal chow diet supplemented with sphingolipids of different origins reduced total hepatic and plasma lipids through improvement of adiponectin signaling and insulin sensitivity [9]. In contrast to the present findings, hepatic cholesterol levels did not change when sphingolipids were administered to those animals. Although total hepatic fatty acids were significantly reduced by sphingolipid supplementation in that study, it was unclear whether this was associated with a reduction in hepatic triglyceride levels. Overall, the effects of sphingolipid supplementation on individual hepatic fatty acids in the prior study were not as significant as the effects observed in the current study.

Gene expression analyses have also shown that dietary sphingolipids suppressed SCD1 expression, which in turn increases insulin sensitivity and activation of acetyl-CoA synthesis (Pdk4), resulting in increased fat catabolism [13]. These results are in contrast to the present study in which we found no consistent effect of increasing doses of SM on plasma glucose, insulin or adiponectin levels (data not shown). It is possible that a longer feeding period might result in subsequent effects on insulin sensitivity. However it is unlikely that the primary effect of dietary SM in the current model is due to an effect on glucose and/or insulin metabolism.

Dietary sphingolipids have previously been reported by Jiang *et al.* to have adverse effects on plasma lipids and atherosclerosis [28]. In their study, LDLr knockout female mice fed a sphingolipid-rich normal chow diet for 3 months had increased levels of plasma cholesterol, SM and non-HDL lipids. These adverse effects clearly contrast with the benefit that was observed in the present study, and could be due to different animal models and/or different diets. We found no change in plasma cholesterol and triglyceride levels in the present study (data not shown), which contrasts with our previous work, where 6 weeks of feeding a SM-containing phospholipid-rich diet significantly reduced plasma cholesterol, triglyceride and phospholipid levels in the same model [13]. We postulate that a longer period of dietary egg SM supplementation may reduce plasma lipid levels in a similar manner. Interestingly, SM supplementation in the present study was associated with a down-regulation of *de novo* ceramide synthesis that correlated with the reduced levels of hepatic ceramide, MHC and DHC species.

In conclusion, the present results demonstrate that dietary SM has potentially beneficial effects on diet-induced hepatic steatosis by reducing intestinal cholesterol absorption. More specifically, these experiments provide evidence that dietary SM, when added to a high-fat diet, potently lowers hepatic cholesterol and triglyceride levels in a dose-dependent manner, most likely through inactivation of LXRα expression and reduced expression of down-stream target genes. Whether dietary SM has the therapeutic potential to ameliorate hepatic steatosis in humans remains to be determined.

## Supporting Information

Table S1 **Sequence of mouse-specific primers used for the gene expression analysis.** Sequences are provided for forward (*F*) and reverse (*R*) primers. ABCA1: ATP binding cassette, sub-family A, member 1; ABCG1: ATP binding cassette, sub-family G, member 1; ABCG5: ATP binding cassette, sub-family G, member 5; ABCG8: ATP binding cassette, sub-family G, member 8; ACC: acetyl-CoA carboxylase; ACO: acetyl-CoA oxidase; CERS2: Ceramide synthase 2; CERS5: Ceramide synthase 5; ChREBP: Carbohydrate response element binding protein; CPT1a: carnitine palmitoyltransferase 1a; CYP4A10: cytochrome P450, family 4, subfamily a, polypeptide 10; DGAT2: diglyceride acyltransferase 2; ELOVL5: elongation of very long chain fatty acids protein 5; ELOVL6: elongation of very long chain fatty acids protein 6; FAS: fatty acid synthase; HMGCoAR: 3-hydroxy-3-methyl-glutaryl-CoA reductase; HMGCoASyn: 3-hydroxy-3-methyl-glutaryl-CoA synthase; LDLr: low density lipoprotein receptor; LXRα: liver X receptor α; MCAD: medium-chain acyl-coenzyme A dehydrogenase; PLTP: Phospholipid transfer protein; PPARα: peroxisome proliferator-activated receptor α; SCD1: stearoyl-CoA desaturase 1; SMPD1: sphingomyelin phosphodiesterase 1; SMPD3: sphingomyelin phosphodiesterase 3; SPTLC2: Serine palmitoyltransferase, long chain base subunit 2; SR-B1: scavenger receptor class B1; SREBP-1c: sterol regulatory element-binding protein 1c; SREBP-2: sterol regulatory element-binding protein 2; VLCAD: very long-chain acyl-coenzyme A dehydrogenase.
(DOCX)

Table S2 **epatic lipid species in mice fed a high-fat diet with or without SM.** Male mice were fed a high-fat diet without (HF) or with HFSM (0.6% wt/wt) for 4 weeks. Hepatic lipid species were quantified by ESI-MS/MS as described in “Methods”. Values represent means ± SEM. Percentage difference between groups is given in italics. Significant difference between HF and HFSM groups were analysed by Student's t-test: * *P*<0.05, † *P_corrected_*<0.05.
(XLS)
