# Supplementary material for: Dietary Sphingomyelin Lowers Hepatic Lipid Levels and Inhibits Intestinal Cholesterol Absorption in High-Fat-Fed Mice
Source: PLoS One. 2013 Feb 7;8(2):e55949. doi: 10.1371/journal.pone.0055949 (PMC3567029; doi:10.1371/journal.pone.0055949)
Supplement: Table S1 — Sequence of mouse-specific primers used for the gene expression analysis. Sequences are provided for forward (F) and reverse (R) primers. ABCA1: ATP binding cassette, sub-family A, member 1; ABCG1: ATP binding cassette, sub-family G, member 1; ABCG5: ATP binding cassette, sub-family G, member 5; ABCG8: ATP binding cassette, sub-family G, member 8; ACC: acetyl-CoA carboxylase; ACO: acetyl-CoA oxidase; CERS2: Ceramide synthase 2; CERS5: Ceramide synthase 5; ChREBP: Carbohydrate response element binding protein; CPT1a: carnitine palmitoyltransferase 1a; CYP4A10: cytochrome P450, family 4, subfamily a, polypeptide 10; DGAT2: diglyceride acyltransferase 2; ELOVL5: elongation of very long chain fatty acids protein 5; ELOVL6: elongation of very long chain fatty acids protein 6; FAS: fatty acid synthase; HMGCoAR: 3-hydroxy-3-methyl-glutaryl-CoA reductase; HMGCoASyn: 3-hydroxy-3-methyl-glutaryl-CoA synthase; LDLr: low density lipoprotein receptor; LXRα: liver X receptor α; MCAD: medium-chain acyl-coenzyme A dehydrogenase; PLTP: Phospholipid transfer protein; PPARα: peroxisome proliferator-activated receptor α; SCD1: stearoyl-CoA desaturase 1; SMPD1: sphingomyelin phosphodiesterase 1; SMPD3: sphingomyelin phosphodiesterase 3; SPTLC2: Serine palmitoyltransferase, long chain base subunit 2; SR-B1: scavenger receptor class B1; SREBP-1c: sterol regulatory element-binding protein 1c; SREBP-2: sterol regulatory element-binding protein 2; VLCAD: very long-chain acyl-coenzyme A dehydrogenase. (DOCX) [file pone.0055949.s001.docx]

| **Supplementary Table 1**  Sequence of mouse-specific primers used for the gene expression analysis | | |
| --- | --- | --- |
| Genes | Forward primers (5’-3’) | Reverse primers (5’-3’) |
| ABCA1 | CGT TTC CGG GAA GTG TCC TA | GCT AGA GAT GAC AAG GAG GAT GGA |
| ABCG1 | CCT TCC TCA GCA TCA TGC G | CCG ATC CCA ATG TGC GA |
| ABCG5 | TCA GGA CCC CAA GGT CAT GAT | AGG CTG GTG GAT GGT GAC AAT |
| ABCG8 | GAC AGC TTC ACA GCC CAC AA | GCC TGA AGA TGT CAG AGC GA |
| ACC | TTC TGA ATG TGG CTA TCA AGA CTG A | TGC TGG GTG AAC TCT CTG AAC A |
| ACO | TTT GTT GTC CCT ATC CGT GAG A | CCG ATA TCC CCA ACA GTG ATG |
| CERS2 | GGA ACA TTT CTA CCA GAC CAG | GAA CCA GGG TTT ATC CAC AG |
| CERS5 | CTC CGT GTT GGC ATT AAA GAC | GGA TCT CCA CAT GCT TTC ACA G |
| ChREBP | CTG GGG ACC TAA ACA GGA GC | GAA GCC ACC CTA TAG CTC CC |
| CPT1a | CCC CAC AAC AAC GG | GCC AGC GCC CGT CAT |
| Cyclophilin | TGG AAG AGC ACC AAG ACA GAC A | TGC CGG AGT CGA CAA TGA T |
| CYP4A10 | AAA GGC TAA TGG TGC GTA CAG ATT | TGG AAC CAT GGC TGT CCA T |
| DGAT2 | CTG GCT GAT AGC TGC TCT CTA CTT C | TGT GAT CTC CTG CCA CCT TTC |
| ELOVL5 | GGT GGC TGT TCT TCC AGA TT | CCC TTC AGG TGG TCT TTC C |
| ELOVL6 | ACA ATG GAC CTG TCA GCA AA | GTA CCA GTG CAG GAA GAT CAG T |
| FAS | ATC CTG GAA CGA GAA CAC GAT CT | AGA GAC GTG TCA CTC CTG GAC TT |
| HMGCoAR | CTT GTG GAA TGC CTT GTG ATT G | AGC CGA AGC AGC ACA TGA T |
| HMGCoASyn | CAT CAC TTA GCC AAC TAT ATT CC | CCA TTC CTT CAT CCA AAC TG |
| LDLr | CTG TGG GCT CCA TAG GCT ATC T | GCG GTC CAG GGT CAT CTT C |
| LXRα | CAG AGC CGA CAG AGC TTC GT | AGC TCG TTC CCC AGC ATT TT |
| MCAD | GCT CGT GAG CAC ATT GAA AA | CAT TGT CCA AAA GCC AAA CC |
| PLTP | AAC TCC ACT TCC AGC CAC ACC | CGC CGA GGC ATT GAT GTA GC |
| PPARα | ATT CGG CTG AAG CTG GTG TAC | CTG GCA TTT GTT CCG GTT CT |
| SCD1 | GAT AGA GCA AGT CCC CGT TG | CCT GCA TTA ACC CCC TTC AC |
| SMPD1 | CCT TCA CAC CCT AAG AAT TGG | TTA TAT GCA CTT TGT CTC CTC G |
| SMPD3 | AGA ATT GTT GGG TAC ATC GC | CTC CAG TTT GTC ATC AGA AGA G |
| SPTLC2 | CGA GAT TTC TTG AGG CAT TGG | TCC CTG TGT ACT TGA ATG ACC |
| SR-B1 | TGG CAT TCA GAG CAG TGT AAC | GAC CCG TTG GCA AAC AGA G |
| SREBP-1c | GAG GAT AGC CAG GTC AAA GC | GCA GGT CAG ACA CAG AAA GG |
| SREBP-2 | GCG TTC TGG AGA CCA TGG A | ACA AAG TTG CTC TGA AAA CAA ATC A |
| VLCAD | TTG TCA ACG AGC AGT TCC TG | AGC CTC AAT GCA CCA GCT AT |

Sequences are provided for forward (*F*) and reverse (*R*) primers. ABCA1: ATP binding cassette, sub-family A, member 1; ABCG1: ATP binding cassette, sub-family G, member 1; ABCG5: ATP binding cassette, sub-family G, member 5; ABCG8: ATP binding cassette, sub-family G, member 8; ACC: acetyl-CoA carboxylase; ACO: acetyl-CoA oxidase; CERS2: Ceramide synthase 2; CERS5: Ceramide synthase 5; ChREBP: Carbohydrate response element binding protein; CPT1a: carnitine palmitoyltransferase 1a; CYP4A10: cytochrome P450, family 4, subfamily a, polypeptide 10; DGAT2: diglyceride acyltransferase 2; ELOVL5: elongation of very long chain fatty acids protein 5; ELOVL6: elongation of very long chain fatty acids protein 6; FAS: fatty acid synthase; HMGCoAR: 3-hydroxy-3-methyl-glutaryl-CoA reductase; HMGCoASyn: 3-hydroxy-3-methyl-glutaryl-CoA synthase; LDLr: low density lipoprotein receptor; LXRα: liver X receptor α; MCAD: medium-chain acyl-coenzyme A dehydrogenase; PLTP: Phospholipid transfer protein; PPARα: peroxisome proliferator-activated receptor α; SCD1: stearoyl-CoA desaturase 1; SMPD1: sphingomyelin phosphodiesterase 1; SMPD3: sphingomyelin phosphodiesterase 3; SPTLC2: Serine palmitoyltransferase, long chain base subunit 2; SR-B1: scavenger receptor class B1; SREBP-1c: sterol regulatory element-binding protein 1c; SREBP-2: sterol regulatory element-binding protein 2; VLCAD: very long-chain acyl-coenzyme A dehydrogenase.
